# Digitalis in Bronchitis

**Published:** 1876-04

**Authors:** 


Digitalis in Bronchitis.—Dr. James Braithwaite writes to the *British Medical Journal* :

Given a patient past the meridian of life, either very stout, or emaciate from previous bad health, and let him, or more commonly her, have a neglected bronchitis, and we have the following class of symptoms produced: A perspiring skin, a poor and quick pulse, urgent dyspnœa, bluish tinge of lips and skin, and respiration accompanied by loud wheezing sounds. The most successful plan of treating these cases, so common during the present severe winter, is the free administration of digitalis, which may be advantageously combined with the compound spirit of ammonia. The digitalis may be given in doses of ten minims of the tincture every two hours.

In these cases it is the heart which is at fault, the right ventricle being gorged with blood, which it is unable to propel through the lungs. The best test of the truth of this theory is its success, for the pulse will be found to become full and slow, and the breathing relieved, directly the digitalis has had time to act on the heart. Its action is sometimes assisted by the judicious administration of a little alcohol. I prefer common gin. If, however, it be found that these cases of commonly called bronchitis are really dependent, for their non-recovery, at least, upon weak hearts, care must be taken as to the administration of stimulants, for they accelerate the action of the heart, and in the end weaken it. In fact, digitalis slackens the speed, and stimulants increase it, so they to some extent contradict each other. I have had some cases lately in which twenty minims of tincture of digitalis were given every two hours for a whole day, and then decreased to ten minims every four or six hours. This medicine will be found to be chiefly valuable when the pulse is weak and rapid. In one old lady about eighty years of age, the effect was remarkable, and in another, seen to-day, the pulse has fallen to 76, with corresponding relief to the other symptoms. In another case, in which the pulse was 130 and very weak, it acted magically, but I am sorry I made no note of the exact rate to which the pulse fell. Good support of all kinds may at the same time be given. I like stimulants less and less in this form of this disease; if you want a horse to go one mile rapidly and well, the spurs may be used, but for a long journey a steady pace is better.

Some patients are more susceptible of digitalis than others, therefore the effects must be watched, and the dose increased or diminished, as necessary.

